# Efficacy of high-intensity interval training versus continuous training on serum myonectin and lipid outcomes in adults with metabolic syndrome: A *post-hoc* analysis of a clinical trial

**DOI:** 10.1371/journal.pone.0307256

**Published:** 2024-07-18

**Authors:** Jorge L. Petro, María Carolina Fragozo-Ramos, Andrés F. Milán, Juan C. Aristizabal, Juan C. Calderón, Jaime Gallo-Villegas

**Affiliations:** 1 Physiology and Biochemistry Research Group-PHYSIS, Faculty of Medicine, University of Antioquia, Medellín, Colombia; 2 Research Group in Physical Activity, Sports and Health Sciences-GICAFS, Universidad de Córdoba, Montería, Colombia; 3 Sports Medicine Postgraduate Program and GRINMADE Research Group, Faculty of Medicine, University of Antioquia, Medellín, Colombia; 4 SICOR Center, Medellín, Colombia; University of Montenegro-Faculty of Medicine, MONTENEGRO

## Abstract

**Background:**

Myonectin is a myokine with potential effects on the lipid metabolism; however, its regulation by exercise in humans remains unclear. We aimed to compare the efficacy of high-intensity interval training low-volume (HIIT) versus moderate-intensity continuous training (MICT) on serum myonectin, serum lipids, appendicular fat and lean mass, and intramuscular lipids in humans.

**Methods:**

Secondary analysis of a controlled, randomized, clinical trial in adults of both sexes with metabolic syndrome, who underwent a supervised, three-times/week, 12-week treadmill program. HIIT (n = 29) consisted of six intervals with one-minute, high-intensity phases at 90% of peak oxygen consumption (VO_2peak_) for a total of 22 min. MICT (n = 31) trained at 60% of VO_2peak_ for 36 min. Serum myonectin was measured using a human enzyme-linked immunosorbent assay. Lipid profile was determined by enzymatic methods and free fatty acids (FFA) were measured by gas chromatography. Fat and lean mass were assessed by dual-energy X-ray absorptiometry. Intramuscular lipids were measured through proton magnetic resonance spectroscopy.

**Results:**

Subjects had a mean age of 50.8±6.0 years and body mass index of 30.6±4.0 kg/m^2^. Compared to MICT, HIIT was not superior at increasing serum myonectin (p = 0.661) or linoleic acid (p = 0.263), reducing palmitic (p = 0.286) or stearic acid (p = 0.350), or improving lipid profile (all p>0.05), appendicular fat mass index ―AFMI― (p = 0.713) or appendicular lean mass percentage ―ALM― (p = 0.810). Compared to baseline, only HIIT significantly increased myonectin (p = 0.042), with a large effect size, although both interventions reduced AFMI and increased ALM with a large effect size. Lipid profile, FFA and intramuscular lipids did not change in any intervention group (p>0.05).

**Conclusions:**

Compared to MICT, HIIT low volume did not demonstrate superiority in improving serum lipids. The fact that both training types reduced AFMI without paralleled significant changes in serum myonectin suggests that this myokine may have a minor effect on short-middle-term exercise-induced fat mobilization.

## Introduction

The metabolic syndrome (MS) is a highly prevalent clinical condition manifested as a combination of cardiometabolic abnormalities such as hypertension, hyperglycemia, atherogenic dyslipidemia and abdominal obesity. Low-grade inflammation, endothelial dysfunction, ectopic fat accumulation (e.g., myosteatosis) and insulin resistance (IR) are key pathophysiologic events underlying those clinical abnormalities [1–5].

Aerobic exercise constitutes a central strategy in the prevention and treatment of the MS. Indeed, different modalities, such as high-intensity interval training (HIIT) and moderate-intensity continuous exercise (MICT) stimulate beneficial changes in the lipid and FFA profiles, and also reduce fat mass (FM) and myosteatosis in patients with overweight, obesity or MS [6–9]. Both types of exercise further modulate the secretion of myokines, mainly peptides or proteins expressed and released by muscle fibers which exert either autocrine, paracrine, or endocrine effects, regulating diverse physiological processes, such as the energy and lipid metabolism. Myokines could then be primary mediators of the effect of exercise on body composition, lipid outcomes and metabolic health [10, 11].

In this context, myonectin has gained attention because it decreases FFA levels in murine models through the stimulation of their uptake by the adipose tissue and liver [12, 13]. Furthermore, high-fat diet-fed mice exhibited decreased expression levels of myonectin in skeletal muscle, but aerobic exercise increased its expression, both in slow-twitch and fast-twitch fibers [12, 14, 15].

The information about the exercise-induced regulation of myonectin in humans, however, is scant. Most of the evidence coincides in that circulating myonectin levels are lower in subjects with MS and related conditions such as type 2 diabetes mellitus (T2DM) and polycystic ovary syndrome [16–18]. Then, if myonectin favors the lipid control in humans and is decreased in the serum of subjects under cardiometabolic disfunction, an exercise intervention which improves lipid outcomes and cardiometabolic risk factors should increase serum myonectin levels. In fact, a previous report showed that an 8-week MICT intervention in obese women increased myonectin [19]. That study, however, did not address the effects of the intervention on the lipid profile, body composition or the intramuscular lipids, hindering any conclusion on the relevance of the myonectin changes to the aerobic exercise-induced changes in the lipid profile, body adiposity, and ectopic intramuscular lipid accumulation. Moreover, since HIIT may generate a greater peripheral stimulus on skeletal muscle [20–22], it could have a greater stimulus on myonectin, something not tested yet. The aim of this study was to compare the efficacy of a 12-week intervention with HIIT versus MICT on serum myonectin, serum lipids, appendicular fat and lean mass, and intramuscular lipids in adults with MS.

## Materials and methods

### Trial design

This is a secondary analysis of a registered, randomized clinical trial (RCT) originally carried out to evaluate the efficacy of high-intensity interval training low volume (HIIT) or moderate-intensity continuous training (MICT) on glucose control variables (NCT03087721) [20, 23]. The current reporting of results follows the CONSORT guidelines [24] (S1 Checklist).

### Ethics approval

This study was approved by the Research Ethics Committees of the IPS-Universitaria healthcare institution (minutes 159 of 2^nd^ March 2021) and the Faculty of Medicine (minutes 005 of 15^th^ April 2021) at University of Antioquia, in Medellín (Colombia), in accordance with the Resolution number 8430 of 1993 issued by the National Ministry of Health of Colombia, and the Ethical Principles for Medical Research Involving Human Subjects outlined in the Declaration of Helsinki in 2008. All patients gave written informed consent.

### Participants

Each participant underwent a complete medical examination. Socio-demographic information and personal and family history were also recorded. Blood pressure and heart rate were measured in the sitting and standing position. For the quantification of physical activity, the validated Global Physical Activity Questionnaire (GPAQ) was used [25, 26]. Subjects of both sexes who were 40 to 60 years old and had MS according to the harmonized definition published in 2009, employing specific definitions of waist circumferences standardized for the Colombian population [27, 28], were included. All participants were recruited from the cardiovascular disease (CVD) risk program of the IPS-Universitaria and the general population in Medellín, Colombia. People with a vegetarian diet, supplemental consumption (e.g., vitamin D_3_), injuries or musculoskeletal diseases that prevented exercise; people in a situation of physical, sensory, and cognitive disability; people with a history of cardiopulmonary disease or acute or chronic inflammatory conditions, cancer, acquired immune deficiency syndrome or T2DM, and pregnant women were excluded.

### Randomization and allocation concealment

The randomization of the participants to either HIIT or MICT groups was performed by the Clinical trial coordination center, external to the researchers, using the minimization method with the MinimPy^®^ v0.3 open software, considering a ratio of 1:1 and the following variables: age (< 50 and ≥ 50 years), sex (male and female), and body mass index (BMI) (< 30 and ≥ 30 kg/m^2^), as described in the original protocol (NCT03087721) [20, 23, 29, 30]. This method does not employ an *a priori* allocation sequence. Instead, it is an adaptive strategy, which used the variance as a measure of the distance of the imbalance between groups, and then assigned the next participant with the "biased coin" method and a base probability of 0.7, in order to reduce that imbalance. The treatment randomization code for each participant was released to the researcher in charge of the allocation (JGV) only after all baseline measurements were completed.

### Interventions

Supervised treadmill sessions were carried out 3 times per week, for 12 weeks, in the cardiac rehabilitation and physiotherapy section at the IPS-Universitaria, Medellín, Colombia, between March 2017 and February 2019, always in the mornings. Briefly, all sessions started with warm-up and finished with cooling-off periods of 3 min at an intensity of 30% of peak oxygen consumption (VO_2peak_). HIIT included six intervals of 1 min of high intensity with a workload of 90% of VO_2peak_ and 2 min with a workload of 50% of VO_2peak_, for a total duration of 22 min. MICT trained at 60% of VO_2peak_ for 30 min, for a total duration of 36 min. All details about the exercise protocols utilized can be found at the Consensus on Exercise Reporting Template (CERT) guidelines previously published [20, 23].

VO_2peak_ was chosen to prescribe exercise as it can be considered the best indicator of a person’s cardiorespiratory capacity and physical fitness. The targeted workloads were delivered in terms of speed and inclination of the treadmill, as previously proposed [31]. VO_2peak_ was determined in a direct ramp ergospirometry treadmill protocol using an Oxycon Delta by Jaeger^®^ (VIASYS Healthcare GmbH, Germany) at the beginning of the study in previously familiarized subjects, as described [20, 23].

### Outcomes

The outcomes assessed in the present *post-hoc* analysis were: serum myonectin, serum lipid profile, appendicular FM index (AFMI), appendicular lean mass percentage (ALM), and intramuscular lipids. All researchers who analyzed the outcomes were blinded to the groups.

#### Serum myonectin levels

For myonectin measurements, frozen serum aliquots were thawed with no extra heat, and 40 μL were poured into the wells of a human myonectin enzyme-linked immunosorbent assay (ELISA) plate (MyBioSource, MBS1600042, San Diego, CA, USA). This kit has a detection range of 0.05–100 ng/mL and a sensitivity of 0.03 ng/mL and showed an intra-assay coefficient of variation (CV) ≤ 4.0% in our laboratory. All plates were processed by a well-trained investigator blinded to the group coding, according to the supplier’s instructions. Optical density was determined on a plate reader (Varioskan Lux, Thermo Scientific, Waltham, MA, USA) at 450 nm.

#### Serum lipids

Fasting venous blood samples were collected before the intervention and between 44–48 h after the last session of the intervention program, using serum separator tubes (BD Vacutainer 367815, USA). The samples were centrifuged at 1300 g for 15 min at room temperature (Rotofix 32, Hettich, Germany) to obtain the serum in which the lipid profile and FFA were measured.

Low-density lipoprotein (LDL-C), high-density lipoprotein (HDL-C) and triglycerides (TG) were determined by enzymatic analyses using a Dimension^®^ equipment RXL Max (Siemens, Germany). Typically, the repeatability of these measurements had a CV of ≤ 3%. The total cholesterol (TC) was calculated using the Friedewald formula [32].

The extraction of FFA was performed by methylation following the Folch method [33]. For this, 40 μL of 50 mg/mL tridecanoic acid and 2 mL of chloroform/methanol (2:1) were added to 100 μL of serum. The mixture was shaken for 1 min and 1 mL of saturated sodium chloride was added. Then, it was centrifuged at 3400 rpm for 7 min and the organic phase was separated and dried in a bath at 90°C. The dry extract was dissolved in 1 mL of hexane and deposited on aminopropyl columns (200 mg). The separation of the FFA was through gas chromatography [34], using a standard (FAME Mix of 37 components: C4-C24, Supelco^®^, USA). An Agilent chromatograph (7890B, USA) with flame ionization detector and TR-CN100 capillary column (60 m x 250 μm x 0.20 μm ID) was used. Helium was used as carrier gas. We analyzed the palmitic 16:0, stearic 18:0 and linoleic 18:2n-6 FFA. Although we also measured arachidonic acid (C20:4n6), nervonic acid (C24:1n9), 11-eicosenoic acid (C20:1n-9c) and adrenic acid (C22:4n-6c), they could not be quantified for all participants, making the number of values available for statistical analysis low. The CV of repeated measurements was ≤ 1%.

#### Fat and lean mass

Participants were weighed on an electronic scale with 0.1 kg accuracy (Omron^®^ HBF-510LA, Omron Healthcare, Inc., Illinois, United States), while wearing minimal clothes. Height was measured to the nearest 0.1 cm with a stadiometer (Seca, Hamburg, Germany). Waist circumference was measured with a fiberglass anthropometric tape at the intermediate point between the lower edge of the last rib and the iliac crest, in the horizontal plane. The BMI was calculated as body mass (kg)/height (m^2^).

Body composition was assessed using Dual-energy X-ray absorptiometry (DXA) with a Discovery Wi DXA system^®^ (Hologic, USA) and the Hologic APEX v4.5.3 software (Hologic, USA), as this technique has shown excellent accuracy in reporting both fat and lean mass [35]. The subjects were examined in the morning under fasting conditions, but well hydrated status, as indicated by a measured urinary density between 1000 and 1025. For this study, AFMI ([arms FM (kg) + legs FM (kg)]/height (m)^2^) and ALM ([appendicular lean mass (kg)/total body mass (kg)] x 100) were considered relevant, because they are associated with an elevated risk of cardiometabolic diseases, disability, and mortality [36, 37]. These appendicular measurements complement our recent report in the same population comparing the effect of HIIT vs MICT on depots of global (total fat mass, fat percentage) and central (android, visceral, gynoid) fat [8].

#### Intramuscular lipids content

The intramuscular lipid content was non-invasively measured through proton magnetic resonance spectroscopy (^1^H-MRS), following international recommendations and protocols previously published [17, 20, 38]. Briefly, for the acquisition, a voxel of 15 x 15 x 35 mm^3^ was placed inside the right vastus lateralis muscle (VLM), due to its relevance to the development of metabolic diseases in humans [39, 40], and the spectra were collected using a flexible coil in a 3T Magnetom Skyra system (Siemens Healthcare, Erlangen, Germany). The spectra were processed with the jMRUI v5.2 free software (http://www.jmrui.eu) [41] to adjust the water reference to 4.7 parts per million (ppm), apodize and subtract peaks from metabolites other than methylenes (CH_2_) from the intramyocellular (IMCL, 1.3 ppm) and the extramyocellular (EMCL, 1.5 ppm). Then, the integrals of the CH_2_ peaks of IMCL and EMCL were quantified. Water amplitude was analyzed in spectra obtained without water suppression. The relaxation times (T_1_ and T_2_) of IMCL-CH_2_ and EMCL-CH_2_ were taken from Valaparla et al. [42] and the absolute concentration of intramuscular lipids per kilograms of wet weight (mmol·kg^−1^ ww) was estimated using the validated equation of Szczepaniak et al. [43, 44]. In our hands, the reliability of the quantification of intramuscular lipids using ^1^H-MRS has a high intraclass correlation coefficient (ICC, 0.98, ―0.97–0.99―).

### Statistical methods

Results are reported as mean ± SD for normally distributed datasets and as median and inter-quartile range (IQR) for skewed ones. The normality of the data and the homogeneity of variances were assessed using the Shapiro-Wilk test and Levene’s test, respectively. Comparisons at baseline were performed with the unpaired Student’s t-test or Mann-Whitney U test for quantitative variables, and Fisher’s exact test for categorical variables.

Intention-to-treat (HIIT, n = 29; MCT, n = 31) analyses were performed. Paired t-test and estimation statistics [45] were used to test the means change within each intervention (pre *vs* post) and then analysis of covariance (ANCOVA) was used to estimate the effect of HIIT compared to MICT (between interventions) on the outcomes included in Table 2. Because ANCOVA has greater statistical power compared to classical methods such as analysis of variance and change-score analysis, it is currently considered the optimum statistical method for the analysis of continuous outcomes in RCT [46]. The results are presented as the adjusted mean difference (95% confidence interval ―CI―). A re-randomization test was performed using permutations for inference purposes. The use of the re-randomization method with permutations is justified by its ability to provide valid statistical inferences and control the frequency of type I errors in situations where conventional methods may not be adequate, such as in clinical trials with small sample sizes where minimization was used, thereby ensuring the integrity and reliability of the results [29, 47, 48].

Effect sizes were estimated with Cohen’s d for repeated measures, using the correction for the correlation of the pre-post values [49]. Myonectin results are shown as natural logarithm (Ln) because of its not normal distribution.

Given that the subsample for the intramuscular lipid measurement was lower (HIIT, n = 9; MICT, n = 12) than the other outcomes, only intragroup comparisons (pre *vs* post) were performed, so they were not included in Table 2. These comparisons were carried out with Wilcoxon test instead of t-test and these analyses can only be considered exploratory.

In this study, the level of statistical significance was set at p<0.05 for all tests. Data analyses were performed using STATA^®^ v14.0 (StataCorp LLC, USA) and SPSS^®^ Statistics v21.0 (IBM, USA).

## Results

### Study participants

**Fig 1:**
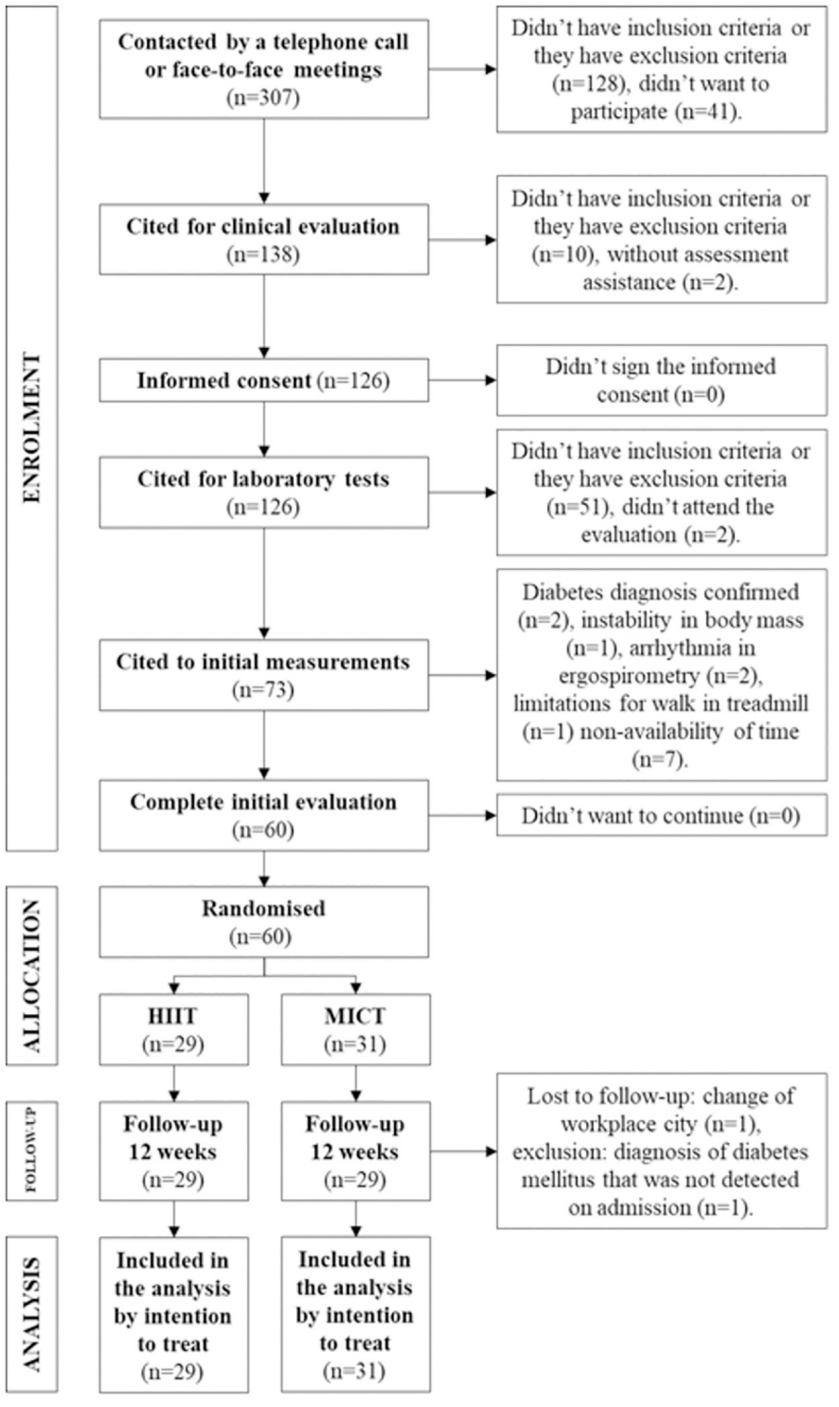
Flow diagram of the clinical trial. HIIT, high-intensity, low-volume interval training; MICT, moderate intensity continuous aerobic training.

Three hundred seven people were contacted for eligibility. One hundred thirty-eight were cited for clinical evaluation, and 60 fulfilled the criteria for participation and were randomly assigned to either the HIIT (n = 29) or MICT (n = 31) groups. One participant was lost to follow-up and one subject was excluded after being diagnosed with T2DM one month after starting participation in the project (Fig 1).

### Baseline characteristics

The mean age of the participants was 50.8 ± 6.0 years, BMI was 30.6 ± 4.0 kg/m^2^ and their median body weight was 75.5 (67.5–84.0) kg. Most participants were female (70%). As expected, the participants were mostly overweight or obese, with dyslipidemia and other CVD risk factors (Table 1, S1 Table). Baseline characteristics were similar between the two groups, except for the use of statins (Table 1).

**Table 1:** Demographics and clinical characteristics of the subjects of the study.

Characteristics	HIIT (n = 29)	MICT (n = 31)	p*
*Demographics*			
Age (years) mean (SD)	51.5 ± 6.0	50.2 ± 6.0	0.409
Female sex, *n* (%)	21 (72.4)	21 (67.7)	0.782
Worker, *n* (%)	16 (55.2)	21 (67.7)	0.408
Unemployed, *n* (%)	12 (41.3)	10 (32.25)	
Retired, *n* (%)	1 (3.4)	0 (0.0)	
*Clinics*			
Hypertension, ^a^ *n* (%)	10 (34.5)	12 (38.7)	0.312
Dyslipidemia, ^b^ *n* (%)	25 (86.2)	25 (80.6)	0.732
Overweight or obese, ^c^ *n* (%)	27 (93.1)	29 (93.5)	1.000
Impaired fasting glycemia, ^d^ *n* (%)	11 (37.9)	16 (51.6)	0.312
*Anthropometrics*			
BMI (kg/m^2^), mean (SD)	30.4 ± 4.0	30.8 ± 4.1	0.731
Waist circumference (cm), median (IQR)	94.0 (89.1–103.2)	94.5 (90.4–102.6)	0.544
Total fat mass (kg), mean (SD)	29.1 ± 6.1	29.7 ± 8.3	0.758
Total lean mass (kg), median (IQR)	40.3 (36.0–51.7)	43.7 (38.8–51.7)	0.318
*Habits*			
Caloric intake, mean (SD)	2117.2 ± 604.8	2118.0 ± 794.6	0.997
Physical activity ≤ 600 MET, *n* (%)	19 (65.5)	15 (48.4)	0.203
Current smoker, *n* (%)	1 (3.4)	3 (9.7)	0.613
*Biochemical*			
Fasting insulin (mUI/L), median (IQR)	14.7 (12.2–18.8)	14.3 (11.2–19.4)	0.663
Fasting glycemia (mg/dL), mean (SD)	98.1 ± 8.8	99.8 ± 8.7	0.454
HbA1c (%), mean (SD)	5.63 ± 0.28	5.72 ± 0.34	0.311
*Medications*			
ACEI or ARA II, *n* (%)	9 (31.0)	11 (35.5)	0.788
Beta-blockers, *n* (%)	4 (13.8)	2 (6.5)	0.417
Calcium antagonists, *n* (%)	2 (6.9)	0 (0.0)	0.229
Diuretics, *n* (%)	5 (17.2)	6 (19.4)	1.000
Aspirin, *n* (%)	2 (6.9)	0 (0.0)	0.229
Statins, *n* (%)	8 (27.6)	2 (6.5)	0.039
Metformin, *n* (%)	2 (6.9)	0 (0.0)	0.229

Values are n (%), mean (SD) or median (IQR). SD, standard deviation; IQR, interquartile range; HIIT, high-intensity, low-volume interval training; MICT, moderate-intensity continuous aerobic training; BMI, Body mass index; BP, blood pressure; HbA1c, glycated hemoglobin; ACEI, Angiotensin-converting enzyme inhibitors; ARAII; Angiotensin II receptor antagonists;

^a^ BP ≥ 140/90 mmHg or treatment with antihypertensive drugs;

^b^ One or more of the following criteria: LDL cholesterol ≥130 mg/dL, triglycerides ≥150 mg/dL or previous diagnosis of dyslipidemia;

^c^ BMI ≥ 25 kg/m^2^;

^d^ fasting blood glucose ≥ 100 mg/dL and < 126 mg/dL;

*p-value of Student’s t-test or Mann-Whitney U-test for quantitative variables and Fisher’s exact test for categorical variables between two groups.

### Outcomes

#### Serum myonectin and serum lipids

**Fig 2:**
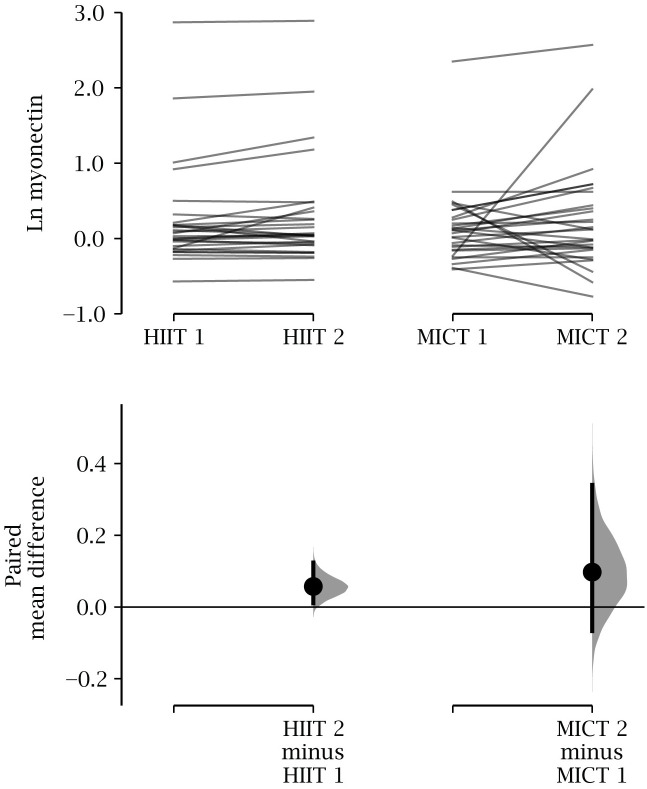
Comparison of the effect of the intervention on serum myonectin. Cumming estimation plot for the paired mean difference of the effect of HIIT or MICT on myonectin. The raw data is plotted on the upper axes. Each paired set of observations (pre and post) is connected by a black line for each intervention type. The lower axes show each paired mean difference (black dots) plotted as a bootstrap sampling distribution (light gray). The 95% CI are depicted as vertical error bars. Paired means difference for HIIT was 0.06 [95% CI 0.01, 0.12], p = 0.042. Paired means difference for MICT was 0.09 [-0.07, 0.34], p = 0.366. HIIT, high-intensity, low-volume interval training; MICT, moderate intensity continuous aerobic training; 95% CI, confidence intervals.

**Fig 3:**
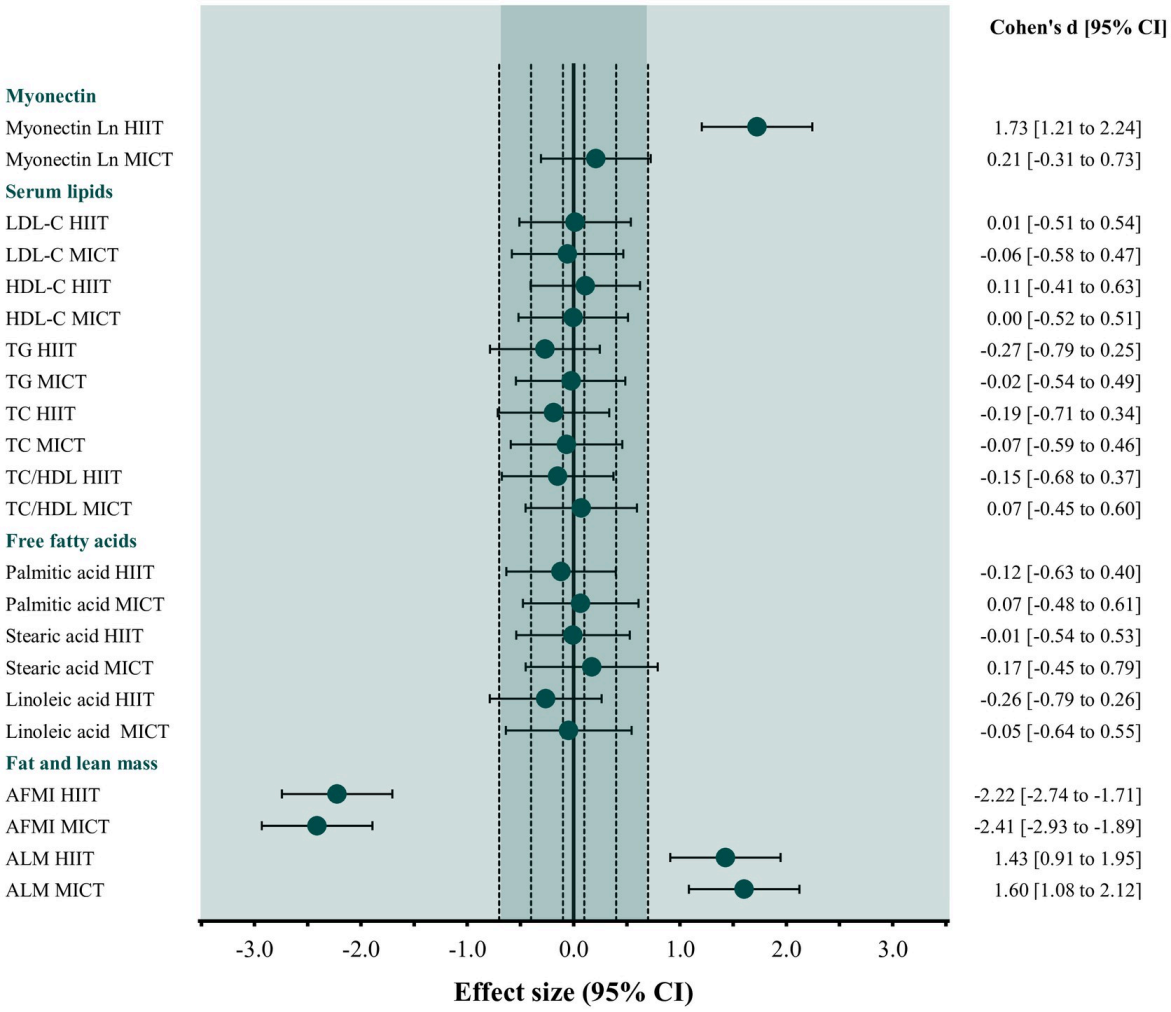
Effect size of the comparison between baseline and after intervention on outcomes in the intention-to-treat analysis (HIIT n = 29, MICT n = 31). Effect size (95% CI) of the comparison between baseline and after 12 weeks of HIIT or MICT intervention on myonectin, serum lipids profile, free fatty acids, fat mass and lean mass. HIIT, high-intensity, low-volume interval training; MICT, moderate intensity continuous aerobic training; LDL-C, low-density lipoprotein; HDL-C, high-density lipoprotein; TG, triglycerides; TC, total cholesterol; TC/HDL, total cholesterol to HDL-C ratio; AFMI, appendicular fat mass index; ALM, appendicular lean mass percentage; 95% CI, confidence intervals.

**Fig 4:**
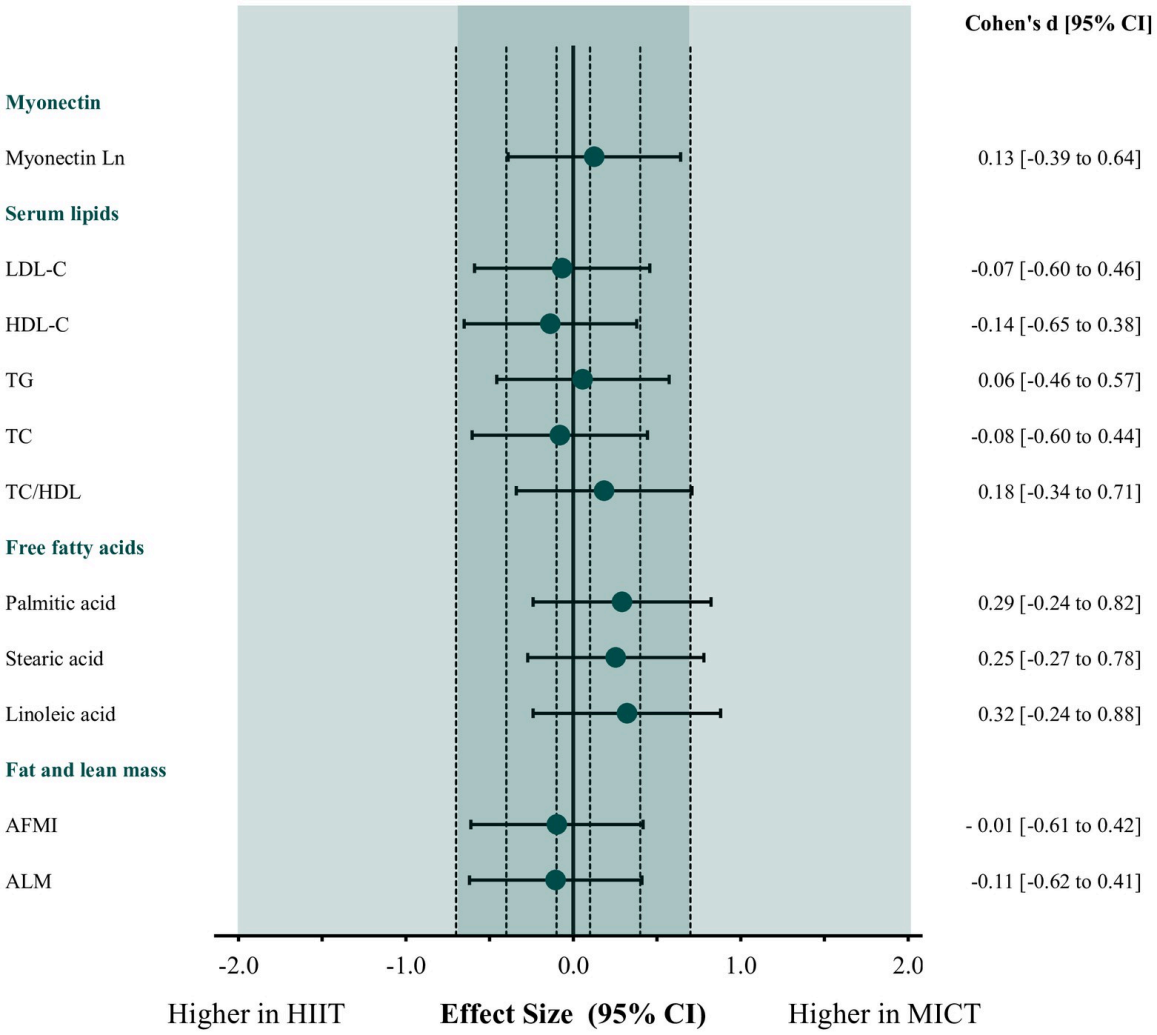
Effect size of the intervention on outcomes in the intention-to-treat analysis (HIIT n = 29, MICT n = 31). Effect size (95% CI) of the comparison between the two training groups on myonectin, serum lipids profile, free fatty acids, fat mass and lean mass after 12 weeks of intervention. HIIT, high-intensity, low-volume interval training; MICT, moderate intensity continuous aerobic training; LDL-C, low-density lipoprotein; HDL-C, high-density lipoprotein; TG, triglycerides; TC, total cholesterol; TC/HDL, total cholesterol to HDL-C ratio; AFMI, appendicular fat mass index; ALM, appendicular lean mass percentage; 95% CI, confidence intervals.

Compared to baseline values, changes in circulating levels of serum myonectin and LDL-C, HDL-C, TG, TC, TC/HDL and the evaluated FFA were not statistically significant in either group (Table 2). However, HIIT showed a trend to increase after the intervention when compared with a paired t-test. Interestingly, the more robust estimation statistics approach revealed a significant change, with a p = 0.042 (Fig 2). Also, Fig 3 confirmed an increase in myonectin with a large effect size only in the HIIT group, with marginal changes for the other serum lipids. Finally, there were no significant differences in myonectin and other circulating lipids between the training groups after the intervention (Table 2, Fig 4).

**Table 2:** Myonectin, serum lipids profile, free fatty acids, fat mass and lean mass results at baseline and after 12 weeks of intervention in the two training groups. Intention-to-treat analysis.

Outcomes	HIIT (n = 29) ^a^				p value^b^	MICT (n = 31) ^a^				p value^b^	HIIT^c^	MICT^c^	Absolute effect of HIIT^d^	95% CI		p value^d^
	Before		After			Before		After			Adjusted mean	Adjusted mean				
	Mean	SD	Mean	SD		Mean	SD	Mean	SD					Lower	Upper	
Myonectin Ln	0.23	0.68	0.29	0.72	0.055	0.14	0.51	0.24	0.70	0.340	0.24	0.29	0.05	-0.17	0.27	0.661
Serum lipids profile																
LDL-C (mg/dL)	149.53	44.57	149.76	49.02	0.970	145.93	59.18	144.80	42.17	0.807	148.18	146.37	-1.80	-16.10	12.48	0.800
HDL-C (mg/dL)	46.37	11.20	47.22	14.22	0.664	43.61	9.43	43.59	10.18	0.985	46.03	44.78	-1.25	-6.12	3.62	0.609
TG (mg/dL)	205.57	91.27	190.45	98.54	0.262	178.84	104.32	177.07	106.25	0.908	181.71	185.81	4.10	-33.76	41.96	0.829
TC (mg/dL)	237.06	56.57	234.85	60.52	0.749	225.96	60.65	224.49	44.59	0.819	230.96	228.38	-2.58	-19.59	14.43	0.762
TC/HDL	5.26	1.14	5.16	1.15	0.622	5.31	1.46	5.37	1.43	0.740	5.18	5.35	0.17	-0.33	0.68	0.492
Free fatty acids (FFA)																
Palmitic acid (μg/dL)	0.19	0.11	0.17	0.09	0.502	0.19	0.10	0.20	0.10	0.699	0.17	0.20	0.02	-0.02	0.07	0.286
Stearic acid (μg/dL)	0.08	0.03	0.08	0.04	0.745	0.08	0.05	0.09	0.05	0.449	0.01	0.01	0.01	-0.01	0.03	0.350
Linoleic acid (μg/dL)	0.13	0.07	0.11	0.05	0.188	0.15	0.13	0.15	0.13	0.810	0.11	0.14	0.03	-0.25	0.09	0.263
Fat mass and lean mass																
AFMI (kg/m^2^)	5.30	1.58	5.20	1.59	0.111	5.49	1.87	5.36	1.84	**0.015**	5.30	5.27	-0.02	-0.18	0.12	0.713
ALM (%)	23.43	3.50	24.72	3.43	0.082	24.41	4.03	24.79	3.97	**0.041**	24.72	24.78	0.05	-0.40	0.52	0.810

^a^ Unadjusted means and SD,

^b^ paired t-test for the change after each intervention,

^c^ means adjusted for age, sex, body mass index and baseline value using ANCOVA,

^d^ difference between HIIT and MICT adjusted means, and ANCOVA P value for the difference between HIIT and MICT adjusted means.

SD, standard deviation; HIIT, high-intensity, low-volume interval training; MICT, moderate intensity continuous aerobic training; CI, confidence interval; ANCOVA, analysis of covariance; LDL-C, low-density lipoprotein; HDL-C, high-density lipoprotein; TG, triglycerides; TC, total cholesterol; AFMI, appendicular fat mass index; ALM, appendicular lean mass percentage.

#### Fat and lean mass

MICT significantly decreased AFMI and both HIIT and MICT reduced it with a large effect size (HIIT, 0.09 kg [95% CI 0.03 to 0.20] Cohen’s d: -2.22 and MICT, 0.10 kg [95% CI 0.02 to 0.22] Cohen’s d: -2.41). Similarly, MICT increased ALM significatively, and both interventions increased it with a large effect size (HIIT, 0.28% [95% CI 0.04 to 0.61] Cohen’s d: 1.43 and MICT, 0.37% [95% CI 0.02 to 0.72] Cohen’s d: 1.60) compared to the baseline (Table 2, Fig 3). There were no differences in AFMI and ALM changes between HIIT and MICT groups after the interventions (Table 2, Fig 4).

#### Intramuscular lipids content

In HIIT, compared to baseline values, IMCL (pre: 12.50 mmol·kg^−1^ ww (4.80–35.12) vs post: 6.96 mmol·kg^−1^ ww (4.99–22.80) (*p* = 0.893)), EMCL (pre: 35.50 mmol·kg^−1^ ww (28.95–56.12) vs post: 35.79 mmol·kg^−1^ ww (25.89–77.95) (*p* = 0.401)) and total intramuscular lipid content (pre: 40.70 mmol·kg^−1^ ww (35.90–48.90) vs post: 38.77 mmol·kg^−1^ ww (28.92–58.55) (*p* = 0.715)) remained unaltered. Similarly, no changes were observed in MICT compared to baseline values in IMCL (pre: 9.15 mmol·kg^−1^ ww (5.50–11.33) vs post: 8.51 mmol·kg^−1^ ww (6.50–9.96) (*p* = 0.401), EMCL (pre: 25.85 mmol·kg^−1^ ww (19.18–34.45) vs post: 20.86 mmol·kg^−1^ ww (14.83–42.95) (*p* = 0.929)) and total intramuscular lipid content (pre: 30.06 mmol·kg^−1^ ww (23.28–42.24) vs post: 25.58 mmol·kg^−1^ ww (20.77–40.91) (*p* = 0.735)). No differences were found between groups at the end of the intervention (p = 304).

## Discussion

The main findings of this study were: (i) HIIT was not superior to MICT in inducing changes in myonectin values or in improving serum lipid profile. Myonectin significantly increased, with a large effect size, only in the HIIT group; (ii) HIIT was not superior to MICT in changing AFMI and ALM, although both interventions improved body composition; (iii) the interventions did not decrease intramuscular lipids.

### The effect of HIIT and MICT on serum myonectin levels

The skeletal muscle is an endocrine organ with a prominent role in the metabolic control and the pathophysiology of cardiometabolic diseases [11]. Myonectin has gained special attention for its potential to mediate the interaction between muscle, exercise, and lipid metabolism [12, 13]. However, the literature regarding this interaction is inconsistent.

One study assigned 80 healthy or overweight/obese women to exercise (n = 34) and control (n = 46) groups. The exercise program comprised three weekly 45-minute sessions of MICT for 8 weeks that included running with 50–70% of maximum heart rate. The authors showed that the serum myonectin level increased significantly in the experimental group [19]. On the other hand, Bahremand and coworkers did not find myonectin changes in a study performed in 30 healthy women that were randomly assigned to CrossFit (n = 16) and concurrent aerobic plus resistance training (CT; n = 14) groups, exercising three sessions per week for 8 weeks [50].

Here, we only found relevant increases in myonectin for the HIIT group, thus confirming an exercise-induced increase in myonectin, but only under some conditions. Interestingly, the work of Bahremand and ours (see the report of results of the primary trial [20]) were effective at modifying cardiovascular (VO_2max_, heart rate at rest), metabolic (FM, glycaemia, homeostatic models of insulin resistance) and muscular variables (lean mass, power output, strength), showing that the exercise interventions were well delivered [20, 50].

These apparently contrasting findings may have different explanations: i) myonectin changes may not be necessary for changes in fat depots but may be necessary for changes in serum lipids, in agreement with findings suggesting that clear changes in the lipid profile in humans can be observed after 16 weeks of training [51], ii) the non-significant changes, even the small effect size increase observed for myonectin in MICT, may reflect early changes sufficient to mobilize lipids from fat depots, which, if augmented or maintained for a long time, may afterwards induce effective changes in serum lipid variables, iii) exercise-induced changes in myonectin are slow, of low magnitude, dependent on the type of intervention and with an effect on other lipid variables on the long term. The last hypothesis seems to be the most supported by the evidence. First, we have shown that lower myonectin levels are associated with an increased android/gynoid ratio [17], and in this work and in a recently published article, with the same population, we saw that several global, central and appendicular FM depots are effectively reduced after a 12-weeks intervention with aerobic exercise [8]. Noticeably, MICT was even superior in reducing android fat mass compared to HIIT [8], but here, the most important change in myonectin was in the HIIT group, likely indicating that myonectin is not a primary mediator of the changes in these fat depots. The larger change in myonectin found in HIIT can be explained because of the larger muscle oxidative metabolic activation, including more activation of fibers type II, under this type of exercise compared with MICT [20, 21]. Since myonectin is produced by both fibers type I and II [12], it is possible that a larger strain imposed during the intervention on fibers type II, and not only on fibers type I, increases the amount of muscle mass releasing myonectin. Instead, the effect of MICT on myonectin seems to be delayed and of a lower magnitude. Second, myonectin levels do not correlate with FFA in subjects with MS under resting conditions [17], therefore, myonectin changes may not be as relevant as initially thought to induce changes in serum lipids. Third, acute, short term physical exertions, or lipid infusions, do not change myonectin in blood [52, 53]. Fourth, the values of circulating myonectin generally found in humans (~0.1–400 ng/mL) [17–19] are up to 4 orders of magnitude lower than those shown to influence lipid metabolism and other functions in different murine and cellular models (~1–5 μg/mL) [12, 54, 55], indicating that in several papers the authors have used pharmacological concentrations of myonectin rather than physiological concentrations to accelerate and potentiate any biological effect.

### The effect of HIIT and MICT on serum lipid profile

Studies comparing the effect of HIIT versus MICT on serum lipids in patients with MS, obesity, and T2DM have shown contradictory results. The positive effects of HIIT alone on LDL have been previously described for Da Silva et al. in a study that enrolled thirty-nine subjects with MS to a 12-weeks exercise intervention [56]. Another study, however, did not show any effect of HIIT on LDL and TC in obese men, even when applied for 16 weeks. Interestingly, the HIIT-MICT alternation rendered better results, improving LDL and TC [51]. In the same sense, Tjønna et al. compared HIIT versus MICT in thirty-two patients with MS and did not find differences between both types of training on LDL, TG and TC [57]. Similarly, other RCT and recent meta-analysis noticed that HIIT was not superior to MICT for modifying LDL, TG, TC or TC/HDL [58, 59]. Together, HIIT is not superior to MICT in improving the lipid profile in subjects with cardiometabolic diseases.

The minor differences found between the studies may be due to the components of the HIIT used (intensity, duration and number of intervals, recovery periods or volume) and duration of the intervention. In addition, is necessary to take in account the time of day of exercise training (morning vs evening). A recently published study, including 25 overweight or obese participants that were allocated to HIIT in the morning (06:30 hours), in the evening (18:30 hours) or no exercise, observed that fasting blood glucose, insulin, TG, TC and LDL-C concentrations decreased only in participants allocated to evening exercise training, as opposed to our protocol, suggesting that the time of day of training would modulate the effect of the exercise on serum metabolomics [60]. In our case, all exercise sessions were performed in the morning, likely reducing their effectiveness.

Studies evaluating the effectiveness of HIIT on FFA compared to MICT in the context of MS suggest that subjects benefit from high volume (higher frequency, longer duration, cointerventions), instead of low volume. For instance, a study conducted in nineteen class II and III obese subjects that were assigned to two weeks of continuous training at the intensity eliciting the maximal fat oxidation (Fat_max_) versus HIIT, showed a decrease of FFA in the group allocated to Fat_max_ (P<0.002) [61]. Also, a decrease in saturated fatty acid (SFA) and n-6 polyunsaturated fatty acid (PUFA) levels with parallel increase of monounsaturated fatty acid (MUFA) and n-3 PUFA was observed after a multidisciplinary intervention that included a calorie-restricted diet (10–40%) and increased continuous physical activity (at least 60 min/5 days a week) during six months [62]. Thus, our intervention seemed to be of lower volume than that required to elicit measurable changes in FFA, something which should be considered when prescribing HIIT modalities to subjects with lipid alterations.

### The effect of HIIT and MICT on body composition and intramuscular lipids content

We recently showed that both MICT and HIIT comparably reduced total FM and gynoid FM, and increased total lean mass in adults with MS, while MICT had additional benefits by reducing the android and the android/gynoid FM ratio [8]. The rather similar beneficial effects of HIIT and MICT, or in any case the no superiority of HIIT, on body composition has been acknowledged in other studies as well [63–65]. In the present study, we extended the analysis to AFMI and ALM and showed the significant beneficial effect of MICT compared to HIIT on AFMI, but HIIT and MICT showed a trend or a significant gain in ALM. This variable has been proposed as a more reliable parameter for the definition of sarcopenic obesity permitting an adequate characterization of the obesity phenotype [66]. Anyhow, both interventions improved body composition with a large effect size. These results highlight that the intervention was well delivered. Moreover, the reduction of AFMI in MICT did not require the increase of myonectin, reinforcing the hypothesis that this myokine may have a minor effect on exercise-induced fat mobilization in humans in the short-middle term.

Although skeletal muscle tissue typically contains small amounts of adipose tissue, several CVD risk factors can lead to an excess of intramuscular deposition of adiposity, in a phenomenon called myosteatosis [67]. Although aerobic exercise of over 10 weeks improves muscle quality in adult populations at risk of developing obesity and sarcopenia-related disability [9], our study failed to show changes in intramuscular lipids content after either HIIT or MICT interventions. Even when our sample size for this outcome was small, we have seen that myonectin does not correlate with intramuscular lipids in a larger sample of subjects with MS [17]. In agreement with the above discussion, we speculate that myonectin may have a minor role in mobilizing intramuscular lipids, however, a larger and longer study should address this specific aim in the future.

### Strengths, limitations, and final remarks

This is one of the few studies comparing the effects of HIIT and MICT on serum myonectin levels and lipid outcomes in people with MS, carrying out the evaluation of body composition and FFA by DXA and gas chromatography respectively, methods with excellent accuracy. Furthermore, we performed robust measures of FFA clinically relevant, closely correlated with metabolic status and the prediction of development of MS [68, 69]. Also, both training groups improved VO_2peak_ around the same magnitude as previously reported [20, 70], indicating a good delivery of the intervention. A *post-hoc* analysis shows a power of 0.9 to detect a pre *vs* post change in myonectin in the HIIT group, and of 0.7 in the MICT group. The power to detect intergroup differences (HIIT vs MICT) in the ANCOVA analysis at the end of the intervention, after multiple adjustments, is 0.75. Although the results presented here were confirmed with a model of re-randomization with permutations (not shown), supporting the validity of the ANCOVA analyses, we cannot fully rule out an error type II when testing the effect of MICT on myonectin. A future work with a larger sample and follow up for over 12 weeks may confirm the hypothesis according to which myonectin responds lower and slower to MICT interventions. Although some participants were on statins, we have shown that they do not change any relationship between myonectin and metabolic outcomes [17], so they are not expected to affect our results. Finally, both interventions were safe as we recently reported [71], helping ensure a low rate of losses.

Although this is the first study in humans evaluating the relationship between myonectin and myosteatosis analyzed by ^1^H-MRS after an exercise intervention, the scope of the results was limited by the restricted number of subjects and the small changes observed, reducing the statistical power for finding differences between the groups. The number of subjects was chosen as a random subsample due to the high costs and demanding sources of the technique, rendering these results thus exploratory, and reinforcing the idea that future works in the field should include a sizeable sample. Ultimately, the results presented here need to be confirmed in further research given the characteristics of the *post-hoc* analysis.

## Conclusions

Compared to MICT, HIIT was not superior at increasing myonectin or improving serum lipid markers. The fact that both training types for 12 weeks reduce fat depots without preceding parallel changes in serum myonectin suggests that this myokine may have a minor effect on exercise-induced fat mobilization in the short-middle term. Long-term MICT stimulation may be necessary to elicit sizeable increases in serum myonectin.

## Supporting information

S1 Checklist CONSORT checklist. (DOCX)

S1 Table. Dataset Contains a dataset of the variables included in this manuscript. (XLSX)
